# B Lymphocytes in Multiple Sclerosis: Bregs and BTLA/CD272 Expressing-CD19+ Lymphocytes Modulate Disease Severity

**DOI:** 10.1038/srep29699

**Published:** 2016-07-14

**Authors:** Federica Piancone, Marina Saresella, Ivana Marventano, Francesca La Rosa, Martina Zoppis, Simone Agostini, Renato Longhi, Domenico Caputo, Laura Mendozzi, Marco Rovaris, Mario Clerici

**Affiliations:** 1 Don C. Gnocchi Foundation, IRCCS, Piazza Morandi, 3, 20121 Milan, Italy; 2 Institute of chemistry of molecular recognition, National Research Council, Milan, Italy; 3 Department of Physiopathology and Transplants, University of Milano, 20100 Milan, Italy

## Abstract

B lymphocytes contribute to the pathogenesis of Multiple Sclerosis (MS) by secreting antibodies and producing cytokines. This latter function was analyzed in myelin olygodendrocyte protein (MOG)-stimulated CD19+ B lymphocytes of 71 MS patients with different disease phenotypes and 40 age-and sex-matched healthy controls (HC). Results showed that: 1) CD19+/TNFα+, CD19+/IL-12+ and CD19+/IFNγ+ lymphocytes are significantly increased in primary progressive (PP) compared to secondary progressive (SP), relapsing-remitting (RR), benign (BE) MS and HC; 2) CD19+/IL-6+ lymphocytes are significantly increased in PP, SP and RR compared to BEMS and HC; and 3) CD19+/IL-13+, CD19+/IL-10+, and CD19+/IL-10+/TGFβ+ (Bregs) B lymphocytes are reduced overall in MS patients compared to HC. B cells expressing BTLA, a receptor whose binding to HVEM inhibits TcR-initiated cytokine production, as well as CD19+/BTLA+/IL-10+ cells were also significantly overall reduced in MS patients compared to HC. Analyses performed in RRMS showed that fingolimod-induced disease remission is associated with a significant increase in Bregs, CD19+/BTLA+, and CD19+/BTLA+/IL-10+ B lymphocytes. B lymphocytes participate to the pathogenesis of MS *via* the secretion of functionally-diverse cytokines that might play a role in determining disease phenotypes. The impairment of Bregs and CD19+/BTLA+ cells, in particular, could play an important pathogenic role in MS.

Multiple sclerosis (MS) is an autoimmune disorder of unknown etiology in which T and B lymphocytes are involved in the initiation and the maintenance of demyelination and axonal damage in the CNS. Much of the research investigating the role of B cells in the pathogenesis of MS focused on these lymphocytes as antibodies producing cells. Myelin-specific antibodies are indeed present in the cerebrospinal fluid, serum, and demyelinating plaques of MS patients^1^,^2^,^3^; there is, however, substantial evidence that B lymphocytes can regulate immune responses by mechanisms other than producing antibodies. Thus, B cells generate cytokines that modulate immune responses^4^, and a number of animal studies show that the selective manipulation of B lymphocytes-produced cytokines can modulate the expression of autoimmune diseases^5^,^6^.

In experimental allergic encephalomyelitis (EAE), in particular, the most widely investigated animal model of MS, interleukin (IL)-10 producing B cells were shown to have an important immunomodulatory role^7^. The ability of CD19+ B cells to secrete IL-10 is greatly reduced as well in patients with MS^8^,^9^,^10^,^11^,^12^, suggesting that the MS-associated inflammatory milieu is at least partly attributable to a defect in IL-10 generation by B lymphocytes. In contrast with what is observed with IL-10, the production of pro-inflammatory cytokines by activated B lymphocytes is increased in MS, and lymphotoxin (LT) and tumor necrosis factor alpha (TNFα) were shown to mediate oligodendrocyte toxicity *in vitro*^13^. This scenario is observed in relapsing-remitting (RR) MS, the most common disease phenotype; in these patients B lymphocytes produce augmented quantities of LT and TNFα and reduced amounts of IL-10 in response to polyclonal stimuli and myelin antigens^9^,^14^,^15^. Notably, *in vitro* results show that antigen-stimulated proliferation of CD4+ and CD8+ T lymphocytes of MS patients is reduced when CD19+ B cells are removed from cultures, possibly as an effect of the diminished secretion of LT and TNFα, cytokines supporting T lymphocytes proliferation, by B cells^14^. Finally, the involvement of B cell in the pathogenesis of MS is supported by the observation that peripheral B cell depletion leads to a rapid decline of disease activity in EAE^16^,^17^.

Upon activation B cells can produce different effector cytokines^8^. B cell activation requires two distinct signals: the first is delivered by antigen binding to B cell receptors (BCR), the second via co-activatory and inhibitory receptors that mostly belong to the B7/CD28 co-receptor family. These molecules regulate numerous checkpoints of immune cells functions, including differentiation, maturation, adhesion, chemotaxis, and the release of soluble factors. B and T lymphocyte attenuator (BTLA or CD272), in particular, is a suppressor molecule belonging to the immunoglobulin superfamily which, like cytotoxic leukocyte antigen-4 (CTLA-4) and programmed death-1 (PD-1), is involved in the inhibition of immune responses. BTLA includes 2 immunoreceptor tyrosine-based inhibitory motifs (ITIM) in its cytoplasmic region^18^, and is expressed on a wide range of hematopoietic cells including T and B lymphocytes, NKT cells, NK cells, macrophages, dendritic cells^19^ and follicular T helper T cells^20^.

BTLA interaction with its ligand, herpes virus entry mediator (HVEM), results in the phosphorylation of tyrosine residues within ITIM, their association with the protein tyrosine phosphatases SHP-1 and SHP-2, and, as a consequence, the inhibition of T-cell activation and the production of anti-inflammatory cytokines including IL-10^19^,^21^,^22^,^23^,^24^. Few data are available on BTLA-expressing B lymphocytes; recent results show that BTLA regulates B cell receptor signaling by reducing the phosphorylation of SYK, B cell linker protein (BLNK), and phospholipase C-γ2^25^. Whereas the biology of BTLA in B lymphocytes in humans has so far been scarcely investigated, more data are available in the animal model. Thus, BTLA-deficient mice are characterized by potent antibody responses, and animals knocked-out for either BTLA or HVEM are susceptible to developing more severe and prolonged forms of EAE^18^.

Data indicating a likely role for B cells and BTLA in the pathogenesis of MS led us to analyze the role of B cells, and in particular of BTLA-expressing B lymphocytes in MS patients affected by different disease phenotypes, and in those whose disease remission is pharmacologically-induced.

## Results

### Expression of pro-inflammatory cytokines by CD19+ B lymphocytes

**Figure 1:**
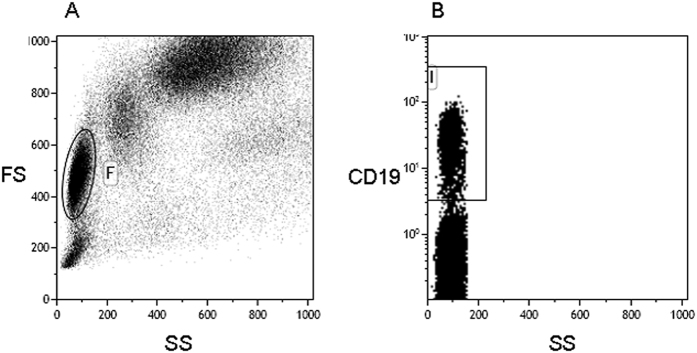
Gating strategies. An initial assessment of lymphocyte population was performed using a forward/sideward scatterplot (FSC/SSC) **(A)**. A first gate was set around the lymphocytes. The lymphocyte population was gated on SSC/CD19-scatterplot identifying B-cells (CD19+) **(B)**. The percentage of TNFα (Fig. 2B), IL-12 (Fig. 2D), IFNγ (Fig. 2F), IL-6 (Fig. 2H), IL-4 (Fig. 3B) and IL-13 (Fig. 3D) CD19+ cells was then calculated using the B cell gate.

**Figure 2:**
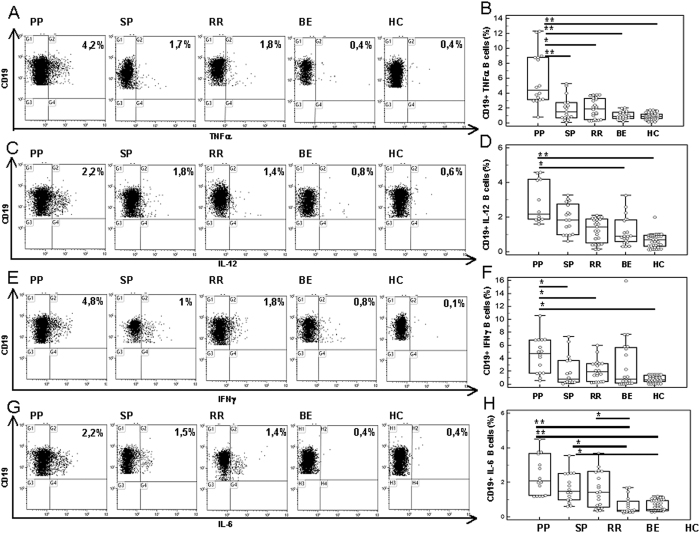
Pro-inflammatory cytokines in MOG-stimulated CD19+ B lymphocytes. Panel B: CD19+/TNFα+ cells; Panel D: CD19+/IL-12+ cells; Panel F: CD19+/IFNγ + cells; Panel H: CD19+/IL-6+ cells. Representative results obtained in MOG-stimulated CD19+ B lymphocytes of PPMS, SPMS, RRMS and BEMS patients, as well as of age-and-sex- matched HC are presented in panels A (TNFα), C (IL-12), E (IFNγ), and G (IL-6); summary results are shown in panels B (TNFα), D (IL-12), F (IFNγ) and H (IL-6). The boxes stretch from the 25th to the 75th percentile; the lines across the boxes indicate the median values; the lines stretching from the boxes indicate extreme values. Statistical significance is shown, *p < 0.05, **p < 0.01, ***p < 0.001, Kruskal- Wallis test.

PBMC of 16 PP, 17 SP, 18 RR, and 20 BEMS patients, as well as of 40 age-and-sex-matched HC were analyzed after being cultured in medium alone (unstimulated) or upon *in vitro* stimulation with either a pool non-antigenic peptides or of MOG peptides. Cytofluorimetric analyses were used to measure the percentage of CD19+ B lymphocytes expressing different pro-inflammatory cytokines: an initial assessment of lymphocyte population was performed using a forward/sideward scatterplot (FSC/SSC) (Fig. 1A). A first gate was set around the lymphocytes. The lymphocyte population was gated on SSC/CD19-scatterplot identifying B-cells (CD19+) (Fig. 1B). Whereas no differences were observed when either unstimulated or non-antigenic-stimulated cells were analyzed, results showed that CD19+ cells expressing TNFα were significantly increased in patients with a diagnosis of either PP, SP and RR multiple sclerosis compared to BEMS individuals and HC, with the highest values being present in PPMS patients. Thus, TNFα- producing CD19+ B cells were significantly augmented in PPMS compared to SPMS (p = 0.01), RRMS (p = 0.017), and BEMS (p = 0.0015) patients, as well as to HC (p = 0.005) (Fig. 2A,B). IL-12-expressing CD19+ B lymphocytes were similarly increased in all groups of MS patients compared to BE and HC, with the differences reaching statistical significance in PPMS (p = 0.02 *vs.* BEMS; p = 0.008 *vs.* HC) (Fig. 2C,D).

Results showed that CD19+ cells expressing IFNγ were significantly increased in patients with a diagnosis of PP multiple sclerosis compared to SP, RR, BEMS individuals and HC (p < 0.005)(Fig. 2E,F).

IL-6-expressing CD19+ B lymphocytes were significantly increased in patients with a diagnosis of either PP, SP and RR multiple sclerosis compared to BEMS individuals and HC, with the highest values being present in PPMS patients. Thus, IL-6- producing CD19+ B cells were significantly augmented in PPMS compared to BEMS (p = 0.006), and HC (p = 0.006), in SPMS compared to BEMS (p = 0.032) and to HC (p = 0.028), and in RRMS compared to BEMS (p = 0.04) (Fig. 2G,H).

### Expression of anti-inflammatory cytokines CD19+ B lymphocytes

**Figure 3:**
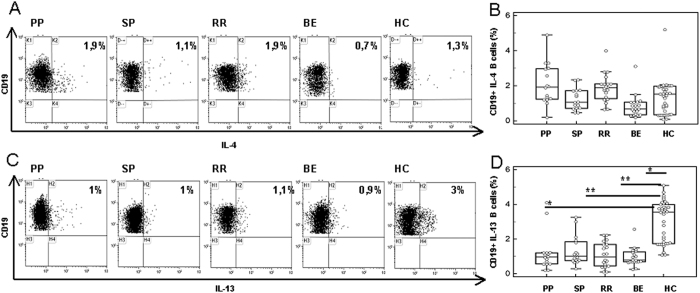
Anti-inflammatory cytokines in MOG-stimulated CD19+ B lymphocytes. Panel A: CD19+/IL-14+ cells; Panel C: CD19+/IL-13+ cells. Representative results obtained in MOG-stimulated CD19+ B lymphocytes of PPMS, SPMS, RRMS and BEMS patients, as well as of age-and-sex- matched HC are presented in panels A (IL-4) and C (IL-13); summary results are shown in panels B (IL-4), and D (IL-13). The boxes stretch from the 25th to the 75th percentile; the lines across the boxes indicate the median values; the lines stretching from the boxes indicate extreme values. Statistical significance is shown, *p < 0.05, **p < 0.01, ***p < 0.001, Kruskal- Wallis test.

Anti-inflammatory cytokines were analyzed next in CD19+ B lymphocytes of all the individuals included in the study. Results showed that, despite the highest percentages of CD19+/IL-4+ being observed in MOG-stimulated cell cultures of HC, B lymphocytes expressing this cytokines were not significantly different in any of the groups examined (Fig. 3A,B). IL-13-expressing B lymphocytes were comparable in MS patients with different disease phenotypes, but were significantly reduced in all groups of MS patients compared to HC (p < 0.05) (Fig. 3C,D).

### CD19+ B regulatory lymphocytes

B regulatory lymphocytes (Bregs) hamper excessive inflammatory responses that occur during autoimmune diseases. Whereas the phenotypic definition of Bregs is still unclear, it is widely accepted that, functionally, Bregs are those B lymphocytes that produce IL-10 and/or TGFβ.

**Figure 4:**
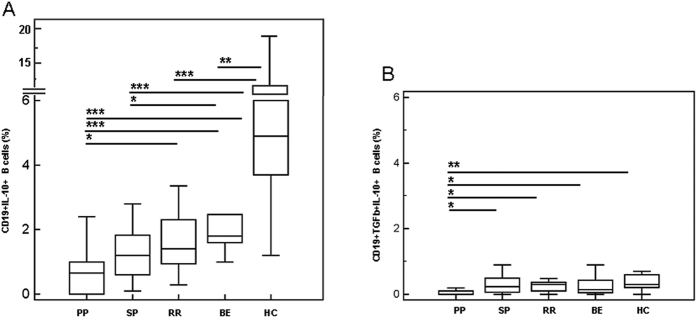
MOG-stimulated CD19+ B regulatory lymphocytes. IL-10-expressing CD19+ B lymphocytes are shown in panel A; IL-10- and TGFβ- coexpressing B cells are displayed in panel B. MOG-stimulated CD19+ B lymphocytes of PPMS, SPMS, RRMS and BEMS patients as well as of age-and-sex- matched HC are shown. The boxes stretch from the 25th to the 75th percentile; the lines across the boxes indicate the median values; the lines stretching from the boxes indicate extreme values. Statistical significance is shown, *p < 0.05, **p < 0.01, ***p < 0.001, Kruskal- Wallis test.

CD19+/IL10+ and CD19+/IL-10+/TGFβ+ B lymphocytes were examined next in all individuals. Results showed that, whereas no differences were seen either in unstimulated or in non-antigenic peptides-stimulated cells, MOG-stimulated Bregs were clearly different amongst diverse groups of MS patients. Thus: 1) CD19+/IL-10+ B lymphocytes were significantly reduced in all groups of patients compared to HC (p < 0.05), with the lowest values been observed in PPMS (p < 0.001) (Fig. 4A); and 2) CD19+/IL-10+/TGFβ+ B lymphocytes were significantly reduced in PPMS patients alone compared to all the other groups of patients (p < 0.05) and HC (p = 0.005) (Fig. 4B).

### BTLA-expressing CD19+ B lymphocytes

**Figure 5:**
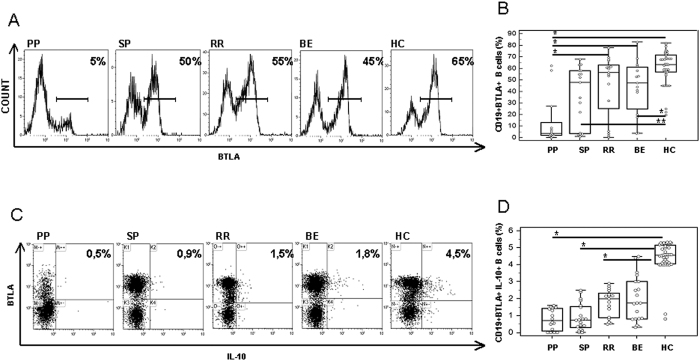
BTLA-expressing CD19+ B lymphocytes. Panel B: CD19+/BTLA+ cells; panel D: CD19+/BTLA+/IL-10+ cells. Representative results obtained in MOG-stimulated CD19 B lymphocytes of PPMS, SPMS, RRMS and BEMS patients, as well as of age-and-sex- matched HC are presented in panels A and C; summary results are shown in panels B and D. The boxes stretch from the 25th to the 75th percentile; the lines across the boxes indicate the median values; the lines stretching from the boxes indicate extreme values. Statistical significance is shown, *p < 0.05, **p < 0.01, ***p < 0.001, Kruskal- Wallis test.

BTLA is a molecule belonging to the immunoglobulin superfamily that is involved in the inhibition of immune responses and plays a role in the pathogenesis of EAE. BTLA-expressing CD19+ B lymphocytes were examined in all individuals. Whereas, once again, no differences were observed either in unstimulated or in control peptides-stimulated cell cultures, results indicated that MOG-stimulated BTLA- expressing CD19+ B lymphocytes were significantly reduced in MS patients compared to HC (p < 0.05). Once again data obtained in PPMS individuals stood out, as the lowest percentages of BTLA-expressing CD19+ B lymphocytes were detected in these patients (p < 0.001 *vs.* HC). Thus, in PPMS, BTLA-expressing B cells were significantly lower than in RRMS (p = 0.03) and in BEMS (p = 0.011) individuals (Fig. 5A,B).

Finally, MOG-stimulated and BTLA-expressing CD19+ B lymphocytes were functionally characterized by analyzing the coexpression of BTLA and IL-10. Results showed that CD19+/BTLA+/IL-10+ cells were overall significantly reduced in MS compared to HC (p < 0.05), with, the lowest percentages being observed in PPMS and SPMS patients (Fig. 5C,D).

### Fingolimod-induced disease remission

**Figure 6:**
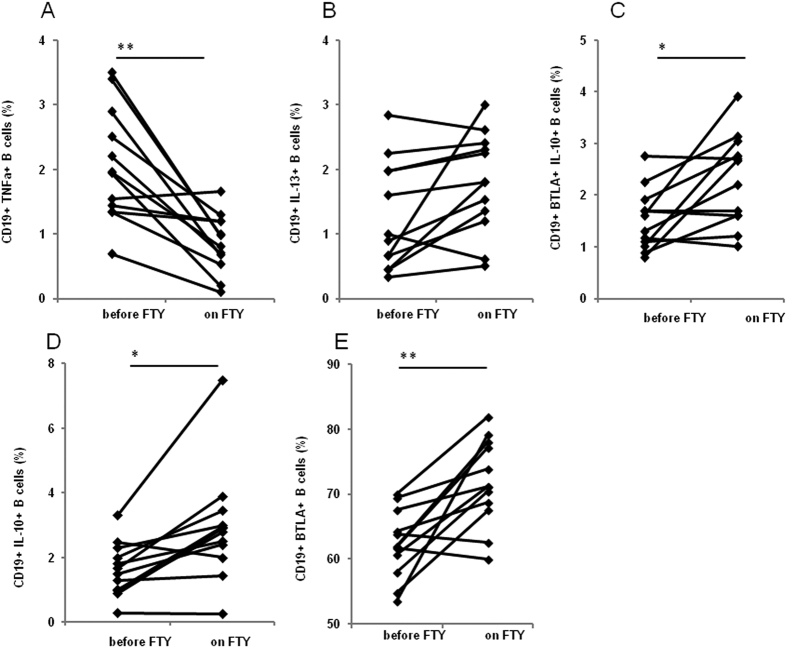
Effect of Fingolimod (FTY) treatment in B lymphocytes in MS. TNFα- (**A**), IL-13- (**B**), BTLA and IL-10 (**C**), IL-10- (**D**) and BTLA- (**E**) expression within the CD19 B cell population before onset of fingolimod treatment (before FTY) and after 1 month of fingolimod-treatment (on FTY) in RRMS patients. Statistical significance is shown, *p < 0.05, **p < 0.01, Kruskal- Wallis test.

Fingolimod (FTY) is an immunomodulator agent directed against the sphingosine-1-phosphate receptor that is approved for the treatment of MS; the beneficial effects of fingolimod are believed to be due to its ability to sequester lymphocytes inside lymph nodes. The effect of fingolimod was evaluated twice in 12 RRMS patients: as soon as disease relapse became clinically and MRI evident, and after clinical and MRI remission was achieved. Results showed that, whereas no differences were seen in CD19+/IL12+ and CD19+/IL4+ lymphocytes (data not shown), CD19+/TNFα+ lymphocytes were reduced (p < 0.001) whilst CD19+/IL-13+, CD19+/IL-10+ Bregs (p = 0.014), CD19+/BTLA+ (p = 0.002), and CD19+/BTLA+/IL-10+ B lymphocytes (p = 0.015) were increased in concomitance with fingolimod-associated disease remission (Fig. 6).

### IL-10 and TNFa mRNA expression by Real-Time PCR

**Figure 7:**
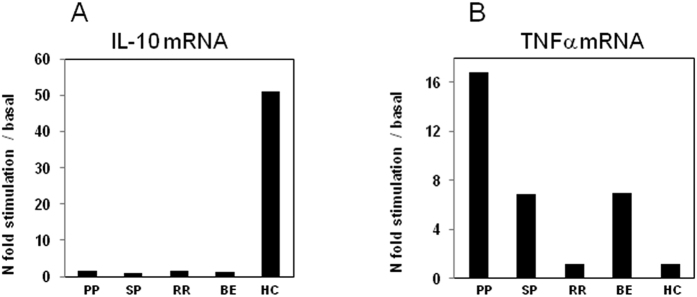
mRNA expression by Real-Time PCR. Single Real-Time PCR results obtained in MOG- stimulated PBMCs of individuals with a diagnosis of PPMS, SPMS, RRMS and BEMS and of age- and sex-matched Healthy Controls (HC). IL-10 is shown in panel A; TNFα in panel B. The results are shown as fold-change expression from the un-stimulated samples. Gene expression was calculated relative to GAPDH housekeeping gene. Summary results are shown in the bar graphs.

Cytokine specific mRNA was finally evaluated by qPCR in MOG-stimulated cells of MS patients and HC. Results showed that: 1) the expression of IL-10 mRNA was increased in PBMCs of HC alone compared to patients with a diagnosis of PP, SP, RR and BE multiple sclerosis (Fig. 7A); and 2) TNFα mRNA was augmented only in PBMC of PPMS patients compared to SPMS, RRMS, BEMS and HC (Fig. 7B).

## Discussion

The role of B cells in autoimmune diseases is still only partially understood despite the well known literature linking linking autoantibodies to autoimmunity^26^. MS, in particular, is characterized by the presence of oligoclonal serum and CSF IgG bands, indicating that B lymphocytes are activated in this disease. The strongest evidence indicating a crucial role for B cells in MS is probably the fact that the use of anti-CD20 drugs (rituximab, ocrelizumab, and ofatumumab), *i.e*. of coumpounds that result in B lymphocytes depletion, reduce MS disease activity and the development of new lesions^16^,^27^,^28^. B lymphocyte depletion, nevertheless, despite improving symptoms of disease does not modulate IgG titers^29^. This observation underlines the complexity of the role played by B lymphocytes in MS, and indicates that these lymphocytes can contribute to the pathogenesis of MS *via* antibody-independent mechanisms. We focused on the ability of B lymphocytes to produce cytokines that are functionally very different, and analyzed the functional subpopulations of B cells in MS individuals with diverse disease phenotypes. Results herein confirm that B lymphocytes are involved in the pathogenesis of MS and indicate that this effect is, at least in part, driven by their ability to produce multiple populations of cytokines. In particular, we observed that different disease phenotypes are associated with the prevalent production of different cytokines by MOG-stimulated immune cells; additionally, we observed that fingolimod-induced disease remission correlates with the modulation of such immune cells.

B cells can be functionally subdivided on the basis of their cytokine profiles; B cells differentiating in the presence of Th1-type cytokines secrete the pro-inflammatory cytokines IFN-γ, IL-12, TNFα and IL-6^30^; B cells that differentiate in the presence of Th2-type cytokines will produce IL-4 and IL-13^31^. Finally regulatory B cells (Bregs) are characterized by the generation of IL-10 and/or TGFβ^32^. We observed that these functional B cell subpopulations are differently distributed in MS patients with active or quiescent disease. Thus, inflammatory B lymphocytes that produce TNFα, IL-12, and IFNγ dominated the immune response in PPMS, a condition characterized by steady worsening of neurologic functioning without distinct relapses or periods of remission. Pro-inflammatory cytokines-secreting B lymphocytes might damage the somatodendritic compartment of neurons in PPMS through the release of molecules acting on synapses^33^. Notably, pro-inflammatory cytokines, such as TNFα, also affect excitatory synaptic transmission, resulting in neurotoxicity *in vitro*^34^. This inflammatory microenvironment is likely maintained in PPMS by the IL-12 produced by MOG-stimulated CD19 B cells, as this cytokine was shown to play an important role in Th1 cells expansion in CNS autoimmune diseases, including MS^35^. MOG-stimulated B cells from PPMS patients secreted increased levels of IL-6 as well compared with B cells from BEMS and controls. IL-6 was shown to exacerbate MS inflammation, Thus, in a murine model of MS, the therapeutic effect of B cell depletion was indeed demonstrated to be mediated by ablation of IL-6 producing B cells^36^. At the opposite end of the spectrum, the frequency of anti-inflammatory cytokines-secreting B lymphocytes was significantly higher in BEMS patients, in whom an absent or minimal neurological impairment is detected, compared to all other disease phenotypes. Notably, B lymphocytes subpopulations in BEMS individuals seem to be characterized by the presence of Breg cells as well as of BTLA-expressing B lymphocytes.

Breg cells are known to suppress immune responses in a variety of murine models of autoimmunity and inflammation^37^; conversely the absence/down-modulation of these cells exacerbate disease symptoms in EAE^5^,^38^,^39^,^40^. In animal models, three studies have shown that Breg cells suppress inflammation upon IL-10 production in colitis^41^, EAE^5^, and arthritis^6^. In addition to expressing IL-10, Breg cells produce other immune-regulatory cytokines, including transforming growth factor β (TGFβ) and IL-35. Interestingly, recent results indicated that lipopolysaccharide (LPS)-activated Bregs can induce the TGFβ-mediated apoptosis of CD4+^42^ as well as the anergy of CD8+^43^ effector T cells^44^.

To our knowledge these are the first data showing a biological role of Breg cells in MS. Thus, CD19+/IL-10+/TGFβ+ B lymphocytes were indeed significantly reduced in PPMS patients alone, suggesting that, in analogy with what was observed in murine model of EAE (see above), this subpopulation of immune cells plays an important role in controlling the inflammation which characterizes this disease. The impairment of these cells in PPMS alone could justify the steady worsening of neurologic function without distinct relapses or periods of remission.

BTLA, on the other hand, is an inhibitory receptor belonging to the CD28 superfamily that negatively regulates immune responses in synergy with the CTLA-4/B7-1 and PD-1/PD-L1 inhibitory pathways^10^,^45^. The interaction between BTLA and its ligand, HVEM, regulates the reciprocal balance between B cell tolerance and activation^46^, and BTLA-expressing B lymphocytes down regulate the intensity of immune responses. Hence, ligation of BTLA by HVEM decreases B cell proliferation^25^, whereas BTLA triggering reduces IL-6, and TNFα secretion and dampens the expression of costimulatory markers (CD80 and CD86) in human B cells^47^. Notably, in EAE, BTLA-deficient mice are characterized by an earlier disease onset, increased clinical score, and a prolonged disease duration^18^.

Interestingly, even if within MS patients the highest percentages of Bregs and BTLA-expressing B lymphocytes were seen in BEMS, these cells were significantly reduced in BEMS compared to HC. The observation that these lymphocytes are reduced even in BEMS patients, in whom no clinical evidence of disease is seen, indicates that MS deeply alters such inflammation-dampening cells. On the other hand, the fact that these cells are increased in BEMS compared to what is observed in patients with active disease (PPMS and SPMS) indicates that they might be sufficient to allow an immunologic control over disease relapses.

Twelve RRMS patients treated with fingolimod were analyzed longitudinally as well; results showed that fingolimod-induced disease remission associates with increased percentages of IL-10-secreting Bregs and of BTLA-expressing, as well as BTLA-expressing and IL-10 secreting B lymphocytes. The mechanism of action of fingolimod is based on the selective modulation of the egress of lymphocytes from lymph nodes secondarily to its effect on sphingosine 1-phosphate 1 (S1P1)^48^. Treatment with fingolimod locks naïve and central memory T cells within lymph nodes but spares effector memory T cells; this drug also reduces the percentages of circulating Th17 lymphocytes^49^. Three recent publications showed that in fingolimod-treated MS patients: 1) naïve B cells as well as PD1-expressing B cells are increased; 2) circulating B cells produce higher amounts of IL-10 and lower quantities of TNFα; and 3) IL-10-secreting CD38+/CD27-/CD24+/CD5+ Breg cells are augmented^50^,^51^,^52^. Our results confirm and expand these data by showing that, besides Bregs, the peripheral population of BTLA-expressing B lymphocytes is relatively expanded as well by fingolimod.

It could be argued that the frequencies of Bregs and BTLA-expressing B cells are low; it is nevertheless important to underline that we analyzed MOG-stimulated cells. We are thus specifically considering and analyzing only those antigen-specific cells that are suspected to drive the disease. In the light of this consideration and of the literature showing how the analysis of apparently minuscule quantities of antigen-specific cells correlates with clinical endpoints^53^, this critique is not justified. Another critique could stem from the fact that, instead of isolating B lymphocytes prior to antigenic stimulation, we stimulated whole PBMC with MOG. As a result, even if we examined B lymphocytes, the results we describe likely stem from an indirect effect of MOG peptide stimulation on the monocyte and T cells that are included in the cell stimulation cultures. This is an important and rational critique. Nevertheless, we decided to use whole PBMC because we wanted to approach as closely as possible the *in vivo* situation, *i.e.* we wanted to reproduce *in vitro* what goes on in the immune system of patients, where immune responses derive from the interaction between multiple cell types. Our results could finally be criticized because they are based on immunological analyses performed in peripheral blood immune cells. A number of data, however, provide evidence that the brain is invaded by peripherally derived monocytes^54^, possibly being transported in the CNS by the very recently described lymphatic vessels that vascularize the brain^55^. Thus, B cells are commonly found in MS lesions^56^,^57^,^58^ and are probably responsible for the local B cell recruitment and maturation at sites of active demyelination. Finally, it is important to underline that overlapping B-cell repertoires are observed on both sides of the blood-brain barrier, suggesting that still unknown disease-driving immunological stimuli are active not only in the CNS but also in the periphery^59^,^60^.

The administration of an agonistic anti-BTLA mAb in a model of murine cardiac allograft was show to associate with the generation of CD4+ T Treg and long-term survival^61^. Even more recently, the transduction of nanoparticles containing MOG peptide with BTLA into dendritic cells was demonstrated to upregulate IL-10 and TGFβ production in EAE, resulting in a significant reduction of disease severity^52^. Results herein strengthen the suggestion that BTLA-directed therapeutic strategies could be considered as a way to design immunosuppressive approaches to MS and, possibly, other inflammatory diseases.

## Materials and Methods

### Patients and controls

All experimental protocols were approved by Don Gnocchi Foundation and all methods were carried out in accordance with the guidelines of the ethic committee of the Don Gnocchi Foundation. All participants gave informed consent according to a protocol approved by the local ethics of the Don Gnocchi Foundation.

Seventy-one patients affected by MS (16 PPMS, 17 SPMS, 18 RRMS, 20 BEMS) as diagnosed by clinical and laboratory parameters, and followed by the Centro Sclerosi Multipla of the Don Gnocchi Foundation in Milano, Italy, were included in the study.

None of the MS patients had received immunosuppressive drugs in the year prior to the study period. Twelve additional RRMS patients undergoing clinical relapses of the disease were evaluated twice: at the onset of disease relapse and after being treated with fingolimod (0.5 mg/die) and having achieved disease remission as evaluated by clinical and MRI parameters. Patients were examined and blood samples were drawn within 7 days after the onset of the acute episode, and always before initiation of therapy. MRI scans showed enhancing lesions in all these patients; none of them had received immunosuppressive drugs in the three months before the study period.

Finally, 40 sex and age matched healthy controls (HC) were enrolled as well in the study.

Demographic and clinical characteristics of all the subjects enrolled are shown in Table 1.

### Peripheral blood mononuclear cell cultures and stimulation

Thirty milliliters of whole blood were collected in vacutainer tubes containing EDTA (Becton Dickinson & Co., Rutherford, NJ, USA). PBMC were isolated from whole blood by centrifugation against density gradient of ficoll-hypaque lymphocyte separation medium (Organon Teknika Corp., Durham, NC, USA) and washed twice in PBS. Viable leukocytes were determined using a Scepter^TM^ Handheld Automated Cell Counter (Millipore, Boston, MA, USA).

Cells were cultured in RPMI 1640 medium supplemented with 10% human serum AB, 2 mM L-glutamine and 1% penicillin (Invitrogen Ltd, Paisley, UK) alone (unstimulated), or were stimulated with either a pool of non-immunogenic peptides^62^ or with a pool of MOG _35–55_ antigenic peptides (a kind gift of Renato Longhi, Institute of chemistry of molecular recognition, National Research Council, Milan, Italy) (10 μg/ml) at 37 °C in a humidified 5% CO_2_ atmosphere for 24 hours (see 26). In order to block protein secretion, Brefeldin A (10 μg/ml) (Sigma-Aldrich, St. Louis, MO, USA) was added to the cell cultures during the last 6 hours of stimulation.

### Flow cytometric analysis

PBMC that had been cultured were washed with PBS and stained with PE-Cyanin-7 (PC7) anti-human CD19 (mouse IgG1; clone J3.119; Beckman-Coulter, Fullerton, CA) and with APC anti-human CD272 (BTLA) (mouse IgG2a,k; clone MIH26, Biolegend) specific mAbs for 30 minutes at RT in the dark. For intracellular staining, cells were fixed (FIX & PERM Cell Permeabilization kits; Caltag Laboratories) for 15 minutes at room temperature in the dark, washed, resuspended in permeabilization reagent (FIX & PERM Cell Permeabilization kits) and stained with APC anti-human TGFβ (mouse IgG1; clone 9016), PE anti-human IL-10 (mouse IgG; clone 127107), FITC anti-human IL-4 (mouse IgG1; clone 3007), FITC anti-human IL-13 (mouse IgG1; clone 32007), FITC anti-human IFNγ (mouse IgG2b; clone 25723), PE anti-human IL-6 (mouse IgG2b; clone 1936), FITC anti-human IL-12 (mouse IgG; clone 27537) (mAbs produced by R&D Systems) and PE anti-human TNFα (mouse IgG1; clone 188, Beckman-Coulter) specific monoclonal antibodies.

Beckman-Coulter Gallios flow-cytometer equipped with two lasers operating at 488 nm and 638 nm, respectively, interfaced with Gallios software was used for the cytometric acquisition. Cytometric analysis was performed with Kaluza v 1.2.

Two-hundred thousand events were acquired and gated on CD19 expression and Side scatter properties. Data were collected using linear amplifiers for forward and side scatter and logarithmic amplifiers for FL1, FL2, FL4, FL5 and FL6. Isotype controls or single fluorochrome-stained preparations were used to evaluate unspecific staining and for color compensation. Rainbow Calibration Particles (Spherotec, Inc. Lake Forest, IL) were used to standardize results.

Gating strategies: An initial assessment of lymphocyte population was performed using a forward/sideward scatterplot (FSC/SSC) (Fig. 1A). A first gate was set around the lymphocytes. The lymphocyte population was gated on SSC/CD19-scatterplot identifying B-cells (CD19+) (Fig. 1B). The percentage of TNFα (Fig. 2B), IL-12 (Fig. 2D), IFNγ (Fig. 2F), IL-6 (Fig. 2H), IL-4 (Fig. 3B) and IL-13 (Fig. 3D) CD19+ cells was then calculated using the B cell gate.

### RNA extraction and reverse transcription

RNA was extracted from unstimulated or 24 hours MOG-stimulated-PBMCs using the acid guanidium thiocyanate–phenol–chloroform method, dissolved in RNase-free water, and purified from genomic DNA with RNase-free DNase (RQ1 DNase; Promega, Madison, WI). One microgram of RNA was reverse transcribed into first-strand cDNA in a 20 μl final volume containing 1 μM random hexanucleotide primers, 1 μM oligo dT and 200 U Moloney murine leukemia virus reverse transcriptase (Clontech, Palo Alto, CA). cDNA were evaluated for GAPDH expression by Real Time PCR to test RNA quality.

### Real Time Quantitative Reverse Transcription

Real Time quantitative Reverse Transcription PCR (RQPCR) was performed on an ABI Prism 7000 instrument (PE Applied Biosystems, Foster City, CA, USA) with gene specific primers; SybrGreen chemistry was used to confirm the gene expression changes observed by arrays. All primers were cDNA specific and were purchased from Qiagen (Venlo, PB Venlo, The Netherlands). Specific PCR products amplification was detected using the RT2 SYBR Green Fluor with a 25 μl final volume of 12.5 μl RT2 qPCR Mastermix (Qiagen) 10.5 μl H_2_O, 1.0 μl of either diluted template or 1.0 μl RT2 qPCR Primer Assay. Results were expressed as ∆∆Ct and presented as ratios between the target gene and the GAPDH housekeeping mRNA.

### Statistical analysis

Quantitative data were not normally distributed (Shapiro-Wilk test) and are thus summarized as median and interquartile Range (IQR; 25° and 75° percentiles). Comparisons between groups were analyzed to evaluate immunological differences. Kruskal-Wallis analysis of variance was performed for each variable; comparisons among the different groups were made using a two-tailed Mann-Whitney test performed for independent samples. Data analysis was performed using the MEDCALC statistical package (MedCalc Software bvba, Mariakerke Belgium).

## Additional Information

**How to cite this article**: Piancone, F. *et al*. B Lymphocytes in Multiple Sclerosis: Bregs and BTLA/CD272 Expressing-CD19+ Lymphocytes Modulate Disease Severity. *Sci. Rep.* **6**, 29699; doi: 10.1038/srep29699 (2016).

## Figures and Tables

**Table 1:** Demographic and clinical characteristics of the individuals enrolled in the study.

	**PPMS**	**SPMS**	**RRMS**	**BEMS**	**RR FTY**	**HC**
N	16	17	18	20	12	40
Gender (M:F)	10:6	10:7	9:9	14:6	7:5	27:13
Age yrs (range yrs)	50 (37–64)	49 (32–67)	40 (20–59)	45 (36–61)	44 (37–53)	48 (33–62)
Disease duration yrs (range yrs)	20 (6–35)	20 (6–35)	7 (1–29)	21 (15–30)	9.5 (8–14)	—
EDSS (range)	6.5 (4.5–8)	6.5 (4.5–8)	1.5 (1–6)	2.0 (0–3)	4.0 (0–6.5)	—

Data are presented as median values. Abbreviations: MS, multiple sclerosis; PP, primary progressive; SP, secondary progressive; RR, relapsing remitting; BE, benign; RR FTY, relapsing remitting under fingolimod-treatment; HC, healthy controls; EDSS, Kurtzke Expanded Disability Status Scale.
